# Rapid and simple detection of *Listeria monocytogenes* using real closed dumbbell-mediated isothermal amplification

**DOI:** 10.3389/fmicb.2025.1596797

**Published:** 2025-08-19

**Authors:** Yanli Zhang, Xinyao Wu, Yeling Zhong, Xuhan Chen, Fei Guo, Guifang Ouyang, Rui Mao

**Affiliations:** ^1^Department of Hematology, The First Affiliated Hospital of Ningbo University, Ningbo, Zhejiang, China; ^2^Ningbo Institute of Life and Health Industry, University of Chinese Academy of Sciences, Ningbo, Zhejiang, China; ^3^Department of General Surgery (Hepatic, Anal-canal, Gastrointestinal), Ningbo Zhenhai People’s Hospital, Ningbo, Zhejiang, China; ^4^Department of Laboratory Medicine, The First Affiliated Hospital of Ningbo University, Ningbo, Zhejiang, China

**Keywords:** *Listeria monocytogenes*, point of care diagnostic, closed dumbbell mediated isothermal amplification (CDA), real-time fluorescence CDA, rapid and simple, visual CDA

## Abstract

**Introduction:**

*Listeria monocytogenes* (*L. monocytogenes*) is a well-known widespread food-borne pathogen that poses a threat to public health. Suitable detection methods are needed to effectively control and prevent pathogenic *L. monocytogenes* infections.

**Methods:**

This study aimed to develop a novel closed dumbbell-mediated isothermal amplification (CDA)-based assay to achieve rapid and simple detection of *L. monocytogenes.* The newly developed CDA technology is capable of amplifying DNA targets with high sensitivity and specificity. The conserved *hly* gene of *L. monocytogenes* was taken as a target for the establishment of the CDA method. All primers were selected and evaluated by real-time fluorescence monitoring, and endpoint visual judgement was indicated by hydroxy naphthol blue (HNB).

**Results:**

The specificity and sensitivity of this CDA-based diagnostic system were determined after the evaluation of 560 batches of DNA samples. The detection limit of the *L. monocytogenes* O-CDA assay was 1 copy/μl using artificial samples. The results of real-time fluorescence-based O-CDA coupled with melting curves analysis showed that the method would achieve rapid and accurate diagnosis of *L. monocytogenes*, and can be employed as an alternative to qPCR in diagnostic practice. Moreover, the *L. monocytogenes* O-CDA method monitored by real-time fluorescence and endpoint hydroxy naphthol blue (HNB) based colorimetric assay displayed the same sensitivity, specificity, and accuracy, which would be helpful to realize onsite pathogen surveillance.

**Discussion:**

The real-time fluorescence plots and following melting curve analysis-based *L. monocytogenes* O-CDA were suitable for laboratory-based diagnosis. Considering the portable manipulation of the HNB-based colorimetric detection system, our results shed light on its potential application for on-site *L. monocytogenes* surveillance. The developed CDA-based methods are rapid, simple, reliable, and sensitive in several samples, showing potential to manage the task of *L. monocytogenes* monitoring more easily.

## 1 Introduction

*Listeria monocytogenes (L. monocytogenes)*, a Gram-positive, short rod bacterium, non-spore-forming, and facultative anaerobic, is a well-known widespread food-borne pathogen (Buchanan et al., 2017). *L. monocytogenes* remains one of the most significant causes of febrile and diarrhea illness (Lourenco et al., 2017). Many ready-to-eat (RTE) food categories, such as fishery, meat, and milk products, could easily be contaminated by *L. monocytogenes*, posing a threat to public health (Aurora et al., 2009; Amagliani et al., 2021; Hansen et al., 2021). Hence, methods of rapid and accurate on-site pathogen identification could be of importance for the effective treatment and control of *L. monocytogenes* (Priya et al., 2020; Shang et al., 2021). Traditionally, bacterial pathogen diagnosis, including *L. monocytogenes,* mainly relied on “gold standard” methods of specimen collection, selective microbial culture, biochemical staining, and colony identification, which required complicated and long procedures and well-trained operators (Park, 2018). Unfortunately, the standard detection method was complex, laborious, time-consuming, and expensive, only available for a limited species (Valimaa et al., 2015). To achieve specific and sensitive pathogen identification, polymerase chain reaction (PCR)—the most practical molecular-based diagnosis method—was adopted (Labrador et al., 2021). Regretfully, the relatively high cost, time-consuming, equipment demanding, and well-trained technicians brought great inconvenience and inappropriate medication to those undeveloped regions with accidents of high prevalence of pathogenic bacterial infections.

At the same time that PCR is being applied in different fields widely, various thermostable nucleic acid amplification methods have been established as alternatives with the merits of nucleic acid signal accumulation with high efficacy (Zhao et al., 2015). Among these methods, loop-mediated isothermal amplification (LAMP) allowed simple and rapid amplification of ultralow nucleic acid molecular in low-infrastructure areas (Notomi et al., 2000; Tomita et al., 2008; Lee et al., 2019). LAMP has been verified in many nucleic acid detection systems and developed into commercial test kits with advantages of robustness, rapidity, specificity, and sensitivity (Fuertes-Perez et al., 2020; Yao et al., 2020; Koeck et al., 2021; Baek et al., 2022). However, the need for specifically designed four to six primers and non-specific amplification posed challenges for the development of practical LAMP-based detection methods and made further application hard (Kimura et al., 2011; Chang et al., 2013; Newbigging et al., 2019). To break the bottleneck of the LAMP method, various isothermal amplification methods, i.e., polymerase spiral reaction (PSR), cross-priming amplification (CPA), competitive annealing-mediated isothermal amplification (CAMP), helix loop-mediated isothermal amplification (HAMP), were developed and validated (Liu et al., 2015; Gao et al., 2018; Mao et al., 2018a; Mao et al., 2018b; Prasad et al., 2024). Our research team developed closed dumbbell mediated isothermal amplification (CDA) method, exhibited merits of simple primer design process, short core primer, and low requirements of target sequence (Mao et al., 2022). The newly developed CDA has been verified in the rapid identification of *Rickettsia raoultii, Klebsiella pneumoniae,* and *Vibrio parahaemolyticus* (Gui et al., 2022; Zhang et al., 2024a; Zhang et al., 2024b). CDA assay was simpler than other molecular diagnostic technologies without a thermal cycler, expensive recombinase, and a long primer sequence. Particularly, the core primer length needed in CDA was reduced to approximately 30 nts compared to ~ 40 nts, which is applied in the above isothermal amplification methods, i.e., PSR, CPA, CAMP, HAMP, and LAMP reaction, which would lead to a dramatic cost saving in primer expense. To the best of our knowledge, a rapid CDA nucleic acid amplification-based detection of *L. monocytogenes* by real-time fluorescence and endpoint visual monitoring has not been reported.

In this study, the CDA method was adopted to achieve on-site, rapid, and simple detection of *L. monocytogenes*. The key indexes of *L. monocytogenes* CDA assays, i.e., specificity, sensitivity, and detection time, were determined. Moreover, the applications of the CDA method for *L. monocytogenes* diagnosis in artificially contaminated materials and samples collected from refrigerators, which were breeding grounds, were also evaluated. All results suggested that the developed *L. monocytogenes* CDA detection methods hold potential as rapid, accurate, robust, inexpensive tools for point-of-care test (POCT).

## 2 Materials and methods

### 2.1 Reagents and materials

Primers were provided by BGI Biological Engineering Technology and Services Co., Ltd. (Shenzhen, China). *Bst* 2.0 WarmStart DNA polymerase and 10 × ThermoPol reaction buffer were purchased from New England BioLabs (Ipswich, MA, USA). DEPC-treated water, 10 mM deoxynucleotide triphosphates (dATP, dTTP, dGTP, and dCTP) mix, and 2 × Taq PCR Mix were obtained from Sangon Biotech (Sangon, Shanghai, China). DNA purification kit (TaKaRa MiniBEST Universal Genomic DNA Extraction Kit Ver.5.0) and nuclease-free water were purchased from Takara Biotech (Takara, Dalian, China). Eva Green and GelRed were obtained from Biotium (Hayward, CA, USA). Other reagents, unless specified, were obtained from Sigma-Aldrich (St. Louis, MO, USA).

The DNA fragment of the *L. monocytogenes hly* gene sequence (GenBank: CP054846.1) was cloned into a pMV vector to construct a plasmid DNA template, and was provided by BGI Biological Engineering Technology and Services Co., Ltd. (Shenzhen, China). The plasmid template DNA of the *hly* gene served as the positive control for the establishment of the *L. monocytogenes* detection. The copy number of the plasmid was determined using the online DNA Copy Number and Dilution Calculator provided by Thermo Fisher Scientific Inc^1^.

### 2.2 Primer design

**Figure 1:**
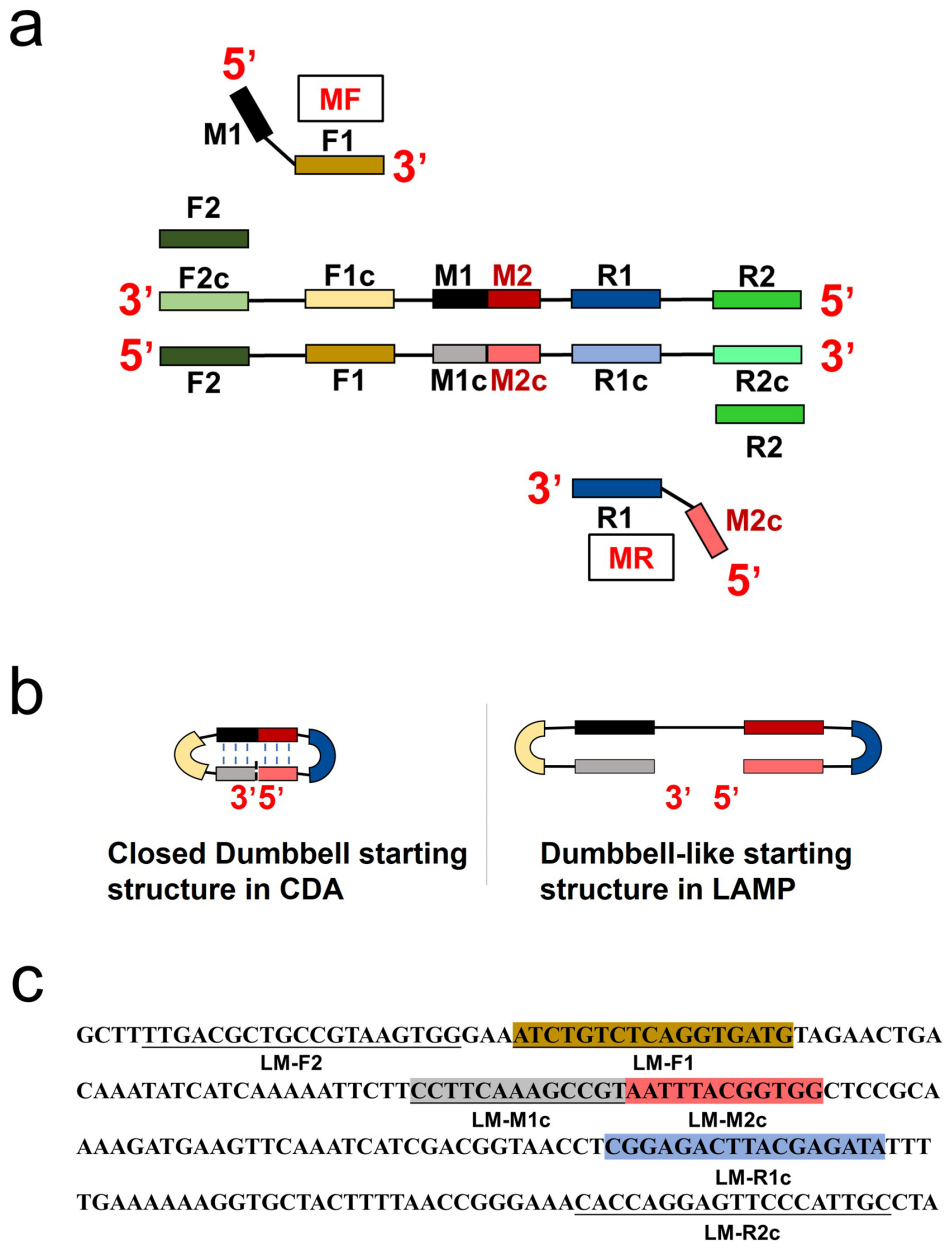
Principle of closed dumbbell-mediated isothermal amplification (CDA) method. **(a)** Overall scheme for primers used in CDA method. **(b)** Comparison of starting structure in CDA and LAMP. **(c)** Partial sequence of the *hly* gene of *L. monocytogenes* (GenBank: CP054846.1) is used for designing the primers of the CDA method.

Compared to conventional LAMP primer design, CDA primers can be obtained from typical PCR primer sequences (typically 18 ~ 24 nts) of the target region. In this study, the scheme for CDA primer design is shown in Figure 1a. The sequence of F1, R1, and M was short for the “forward 1,” “reverse 1,” and “middle,” respectively. The sites with lowercase “c” represented “complementary” sites. An inner sequence M (typically 20 ~ 24 nts) between F1c and R1 was divided into two parts, M1 and M2 (typically 10 ~ 12 nts). Given this structure, the primers of MF and MR were designed as follows. MF contained the sequence M1c and the sequence F1, and MR contained the sequence M2 and R1. The CDA primers were designed based on the *hly* gene of *L. monocytogenes*, using DNAMAN version 8.0 (Figure 1c). For further optimization, outer primers (F2 and R2) were also designed to accelerate CDA reaction (O-CDA) outside of the F1 and R1. The locations of all CDA primers used in this study were labeled with different colors, as shown in Figure 1c. The sequences of primers are listed in Table 1 and Supplementary Table 1.

**Table 1:** Primer sequences of O-CDA and PCR targeting at *Listeria monocytogenes hly* gene (GenBank: CP054846.1 and other confirmed sequences).

Target	Method	Primer	Sequence (5′ → 3′)
*Listeria monocytogenes hly* gene, GenBank: CP054846.1	CDA	LM-MF	ACGGCTTTGAAGG-ATCTGTCTCAGGTGATG
		LM-MR	AATTTACGGTGG-TATCTCGTAAGTCTCCG
		LM-F2	TTGACGCTGCCGTAAGTGG
		LM-R2	GCAATGGGAACTCCTGGTG
	PCR	LM-F	ATCTGTCTCAGGTGATG
		LM-R	TATCTCGTAAGTCTCCG

### 2.3 CDA reaction

The CDA assays were carried out in 25 μL reaction mixtures: 8 U *Bst* 2.0 WarmStart DNA polymerase, 2.5 μL 10 × ThermoPol reaction buffer, 1 M betaine, 6 mM MgSO_4_, 1.4 mM of each dNTP, 1.6 μM of MF and MR, and an appropriate amount of nucleic acid template. DEPC-treated water was used to adjust the volume to 25 μL. The O-CDA reaction contained another pair of primers, F2 (0.2 μM) and R2 (0.2 μM). CDA reactions not containing targeted nucleic acid sequences were set as negative control (NC). The reactions were generally performed at 60 ~ 65°C for 60 min, and then heated at 85°C for 10 min to terminate. SLAN-96 Real-Time PCR detection system (Sansure biotechnology, Changsha, China), CFX96™ Real-Time System (BIO-RAD, California, America), and Q3 Real-Time PCR instrument (Applied Biosystems) were adopted by adding 1 × Eva green for real-time fluorescence monitoring and melting curve analysis of the CDA amplifications.

As similar reaction mixtures were adopted, the CDA amplification also produces insoluble magnesium pyrophosphate compared with LAMP (Goto et al., 2009). Hydroxy naphthol blue (HNB, final concentration 120 μM) was added to indicate Mg^2+^ reduction in the assay described earlier to achieve colorimetric endpoint monitoring of the reaction by naked eye. A positive reaction is indicated by a color change from violet to sky blue, while a negative reaction remains violet in the HNB reaction mixture.

### 2.4 Real-time quantitative PCR reaction

Real-time quantitative PCR (qPCR) assays of the same samples were carried out for a comparison method of the developed *L. monocytogenes* CDA assay. The F1/R1 sequence of the *hly*-CDA assay (F1: 5′- ATCTGTCTCAGGTGATG-3′ and R1: 5′- TATCTCGTAAGTCTCCG −3′, Figure 1 and Table 1) was used to perform PCR reaction in a 50 μL volume, which contained 25 μL of 2 × Taq PCR Mix (Sangon, Shanghai, China), 10 mM of each primer, and DNA samples. Final concentration of 1 × Eva green was added to mixtures to achieve real-time fluorescence monitoring in SLAN-96 Real-Time PCR detection system (Sansure biotechnology, Changsha, China), CFX96™ Real-Time System (BIO-RAD, California, America) or Q3 Real-Time PCR instrument (Applied Biosystems). The PCR reaction was conducted under the conditions as follows: denaturation at 95°C for 2.5 min, annealing at 55°C for 30 s, and extension at 72°C for 20 s, for a total of 35 cycles, for nearly 2 h. Finally, melting curve analysis procedures of PCR products were conducted in the same apparatus.

### 2.5 *L. monocytogenes* samples detection by *hly* CDA method

To investigate the practicability of *hly* CDA method, real-time and endpoint colorimetric detection of *L. monocytogenes* and other pathogens were taken as examples. The developed *L. monocytogenes* O-CDA assays were conducted, only nucleic acid templates were different. Typically, positive controls using the *hly* gene and negative controls using nuclease-free water were essential in the assays. A total 11 *L. monocytogenes* strains of different origins were used in this study (Supplementary Table 2). For bacterial DNA preparation, a single clone of the bacteria was picked from an LB agar plate and cultured at 37°C in LB broth overnight in a shaker incubator (180 rpm). Pasteurized milk (5 mL) and smashed bread (5 g) were artificially contaminated by *L. monocytogenes* standard strain (CVCC 1598) cultivated in sterilized 50 mL corning tubes at room temperature overnight. After incubation or contamination, the samples were collected and extracted using DNA purification kits or easily prepared by boiling and centrifugation. In addition, 8 batch samples collected from refrigerators, which were breeding grounds for *Listeria monocytogenes,* were also tested. All the extracted DNA samples were measured with the values of OD_280_/OD_260_ between 1.85 and 1.93 and stored at −20°C. To evaluate the sensitivity of the CDA method, the 10-fold serial dilutions of *L. monocytogenes* (CVCC 1598) strain, ranging from 1.0 × 10^6^ to 1 copies/μl, were prepared with sterile water.

The specificity of *L. monocytogenes* CDA assay was further confirmed by DNA samples extracted from standard strains of *Listeria innocua* (ATCC 33090), *Candida albicans* (CICC 1965), *Candida tropicalis* (BNCC 186815), *Streptococcus agalactiae* (BNCC 185941), *Bacillus thuringiensis* (BNCC 353357), *Escherichia coli* (CVCC 1491), *Shigella sonnei* (CVCC 3926)*, Vibrio parahaemolyticus* (CGMCC 1.1997), *Salmonella typhimurium* (ATCC 14028), *Staphylococcus aureus* (CGMCC 1.6750), and *Bacillus cereus* (CICC 21261), as listed in Supplementary Table 2. In all, 208 batches of DNA samples (including 40 batches of artificially contaminated samples by the standard strain) from 11 different origins of *L. monocytogenes* strains were used for evaluation of the potential POCT application of the developed *hly* CDA assay.

## 3 Results

### 3.1 Designing and screening of CDA primer for *L. monocytogenes*

**Figure 2:**
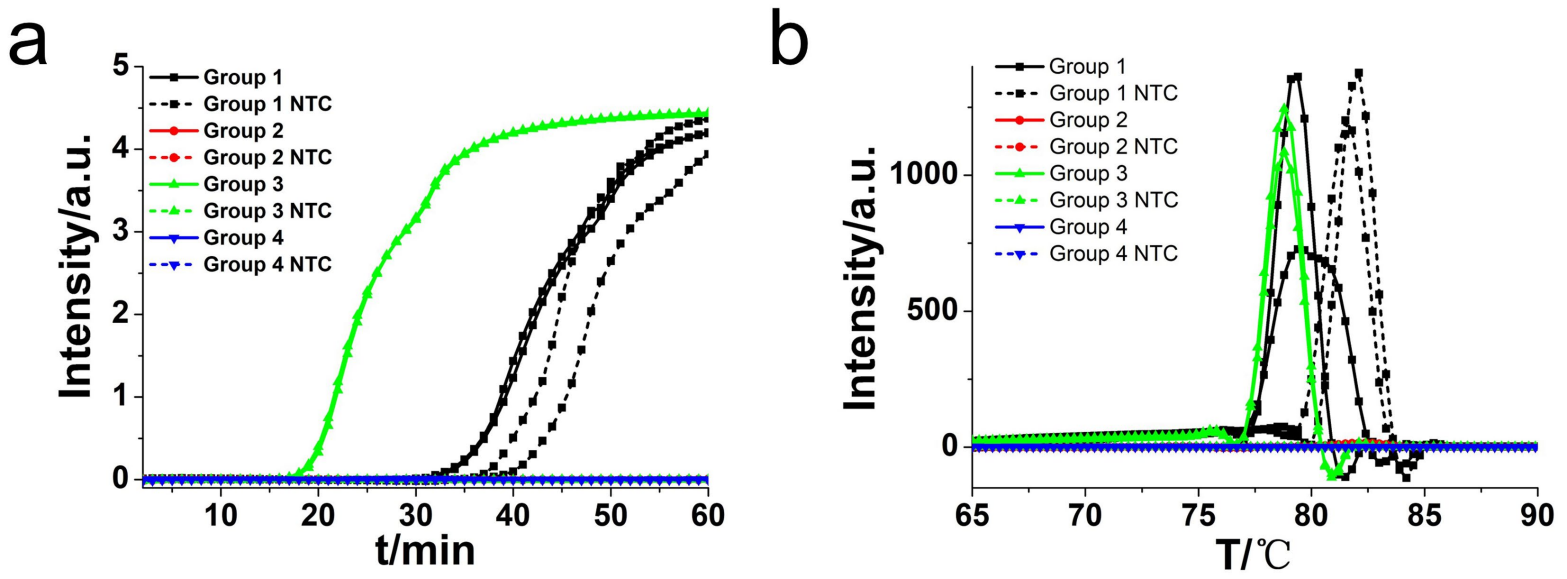
Amplifications of *L. monocytogenes* gene by CDA monitored by real-time PCR carried out at 63°C for 60 min. **(a)** Real-time CDA of different primer groups for *hly* gene (each set 2 positive reactions and 2 negative reactions, no template control: NTC). **(b)** Melting curve analysis of *hly* CDA products by real-time PCR.

**Figure 3:**
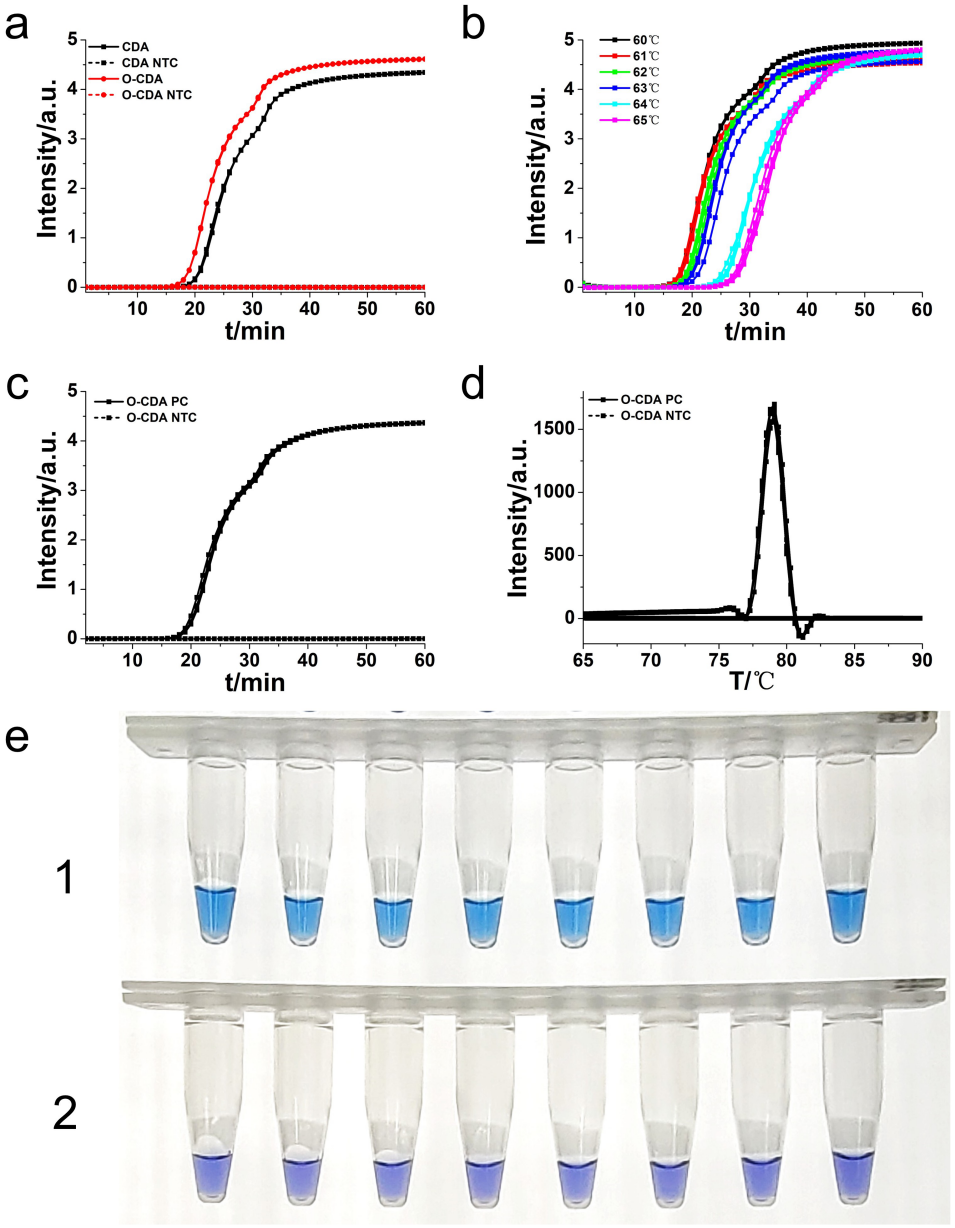
Amplifications of *L. monocytogenes* gene by CDA and O-CDA monitored by real-time PCR and HNB-indicated visualization. **(a)** Amplification plot of real-time CDA and O-CDA for *hly* gene (both set 2 positive reactions and 2 negative reactions). **(b)** Amplification plot of real-time O-CDA for *hly gene* at different temperature (each set 8 positive reactions). **(c)** Repeatability analysis of the O-CDA for hly monitored by real-time PCR at 61°C. **(d)** Melting curve analysis of hly O-CDA products. **(e)** HNB-based *hly*O-CDA reaction of 8 positive samples (line 1, sky blue) and 8 negative samples (line 2, violet). PC: Positive control; NTC: no template control.

Following the CDA primer design criteria, a total of four CDA primer sets for amplification of the *L. monocytogenes* gene were designed and synthesized (Supplementary Table 1). The overall isothermal nucleic acid amplification mechanism of CDA is like LAMP. Instead of the inner primer of FIP and BIP in LAMP, MF and MR in CDA played major roles in initiating self-priming of the starting structure and facilitating synthesis of the target DNA (Figures 1a,b). Like the dumbbell-like structure in LAMP to innate self-primed DNA synthesis, a closed dumbbell structure [5′-M1-R1c-Mc(M1c-M2c)-F1-M2-3′] was formed in the initial amplification phase of CDA reaction, as depicted in Figure 1b. The cycle thresholds (Ct) of the *hly* gene CDA amplification monitored by real-time fluorescence were negatively correlated with the reaction efficiency, as shown in Figure 2. By using this method, the optimum CDA primer set (group 3) with minimum Ct value targeting the *hly* gene was determined and verified. The threshold detection time for 1.0 × 10^7^ copies was 18 min, and the melting temperature for the amplification products was 78.20°C, and no non-specific amplification was observed. Both real-time amplification curve and melting curve indicated a good repeatability (4 repeats) and stability of the *hly* CDA method (Figure 3a).

### 3.2 Optimization of CDA primer set for *L. monocytogenes*

To achieve higher efficiency of CDA reaction, outer primers (F2 and R2) of the *hly* gene were designed and evaluated based on the selected primer set (group 3). The addition of basic primers (MF and MR) and outer primers to the CDA assay was termed as O-CDA reaction. As shown in Figure 3a, the threshold time was shortened by 2 min in the detection of *L. monocytogenes* (1.0 × 10^7^ copies) by O-CDA. The results proved that the reaction efficiency of the CDA method for *hly* gene amplification was improved by the addition of acceleration primers. After determination of the outer primer, the reaction temperatures of the *hly* O-CDA assay were optimized by incubating at 60°C, 61°C, 62°C, 63°C, 64°C, and 65°C, respectively. As shown in Figure 3b, the optimum reaction temperature was set at 61°C to ensure positive detection in each four replicates.

To further evaluate the repeatability of the developed assay, 1.0 × 10^7^ copies of the *hly* gene were amplified by the developed O-CDA method (both set eight positive and negative replicates independently). The real-time amplification curve (Figure 3c) and melting curve (Figure 3d) of *hly*-O-CDA reactions indicated a good repeatability of the established method. Moreover, the melting curves in Figures 2B, 3d were similar, indicating the acceleration effect and no non-specific amplification after the addition of outer primers.

Furthermore, as shown in Figure 3e, eight positive and negative *hly* O-CDA reactions exhibited by HNB were carried out at 61°C for 60 min. The results of lines 1 and 2 indicated that the results of *hly* CDA assays could be visualized by the naked eye (100% success), indicating potential for on-site detection of *L. monocytogenes*.

### 3.3 Sensitivity and specificity assay for O-CDA and PCR of *L. monocytogenes*

**Figure 4:**
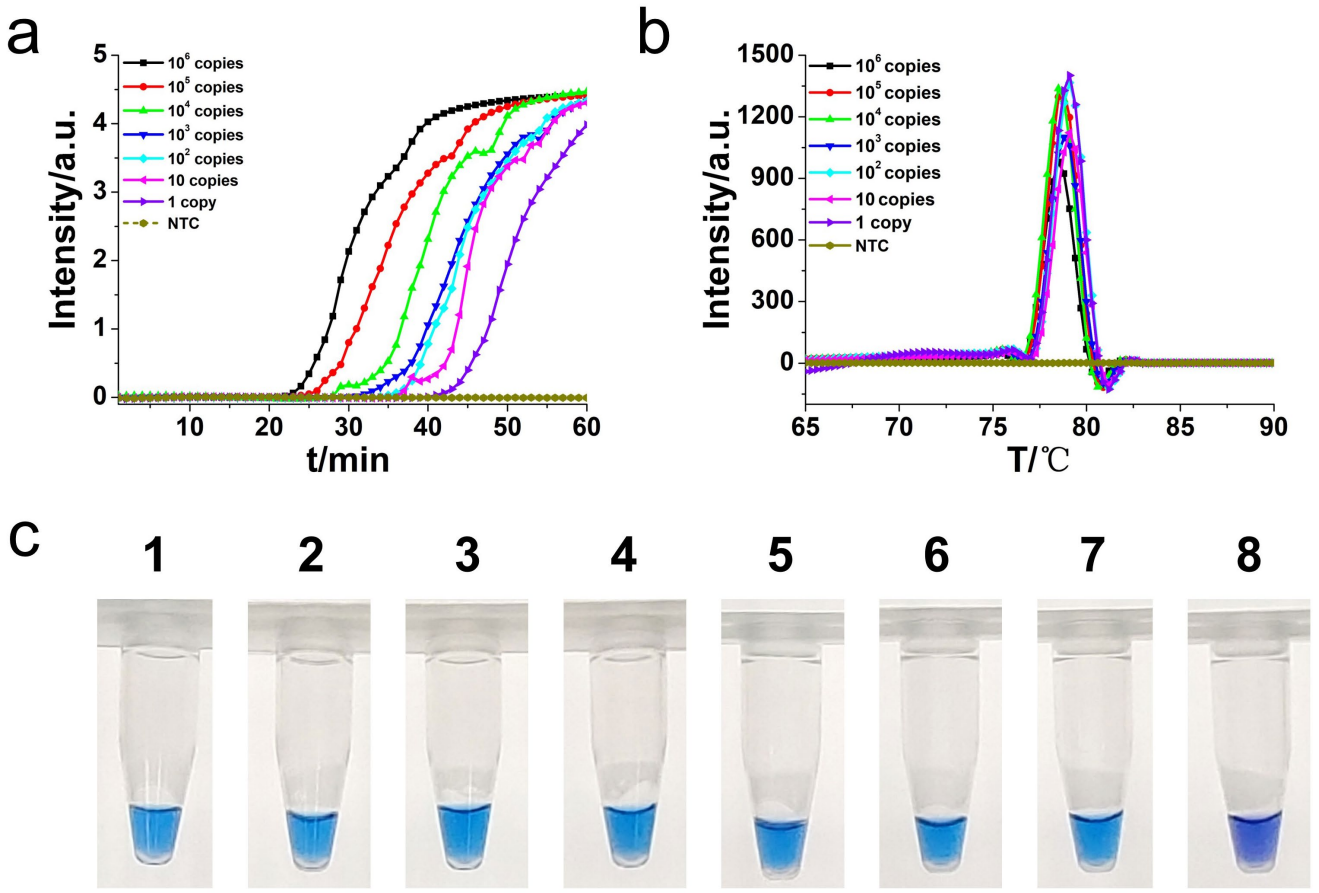
Sensitivity analysis of the *L. monocytogenes* gene O-CDA method by real-time and visual approaches. **(a)** O-CDA amplification was monitored by real-time PCR every 1 min at different concentrations of DNA. Reaction was performed at 61°C for 60 min. **(b)** Melting curve analysis of *hly* O-CDA products at different concentrations of DNA. **(c)** Sensitivity analysis of *hly* detection by visual O-CDA. The DNA concentrations were as follows: 10^6^ copies/μl, 10^5^ copies/μl, 10^4^ copies/μl, 10^3^ copies/μl, 10^2^ copies/μL, 10 copies/μl, 1 copy/μl, and negative control (no template control, NTC).

**Figure 5:**
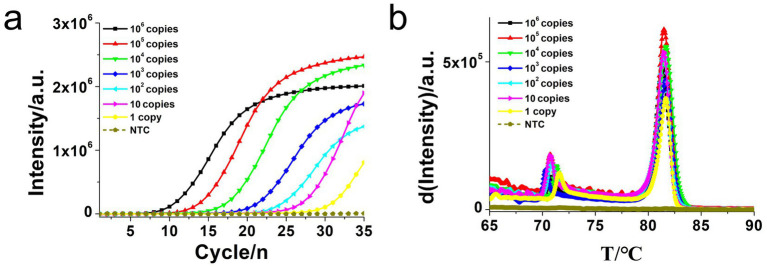
Real-time quantitative PCR (qPCR) assay for *L. monocytogenes* gene detection. **(a)** Real-time amplification plot. **(b)** Melting curve analysis of *hly* qPCR products at different concentrations of DNA. The DNA concentrations were as follows: 10^6^ copies/μl, 10^5^ copies/μl, 10^4^ copies/μl, 10^3^ copies/μl, 10^2^ copies/μl, 10 copies/μl, and negative control (no template control, NTC).

To determine the sensitivity of the developed *hly* O-CDA assay, 10-fold serial dilutions of *L. monocytogenes* genomic DNA concentrations ranging from 10^6^ copies/μl to 1 copy/μl were tested. Identical *L. monocytogenes* genomic DNA was used to compare efficiency between O-CDA and qPCR. As indicated by the real-time fluorescence curve and color change of the reaction products, the lowest limit of *L. monocytogenes* detection was determined to be 1 copy/μl DNA for the O-CDA assay (Figures 4a,c). In comparison, the detection limit of the real-time quantitative PCR (qPCR) assay using the specific primers F1 and R1 was 1 copy/μl DNA (Figure 5A). Furthermore, the melting curve analysis by both real-time fluorescence O-CDA and qPCR for *L. monocytogenes* detection showed no difference in the Tm values observed for all template concentrations (Figures 4b, 5b). The results indicated that the O-CDA assay had a wider dynamic range with similar detection sensitivity compared with that of the qPCR approach for *L. monocytogenes* detection.

**Figure 6:**
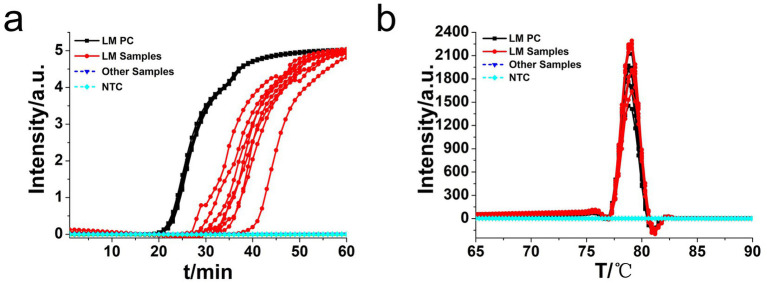
Real-time *L. monocytogenes* O-CDA assay. Real-time O-CDA reactions were carried out at 61°C for 60 min. **(a)** Real-time O-CDA for *hly* gene positive controls (*hly* PC), different *L. monocytogenes* samples, other extracted DNA samples (*Listeria innocua, Escherichia coli*, *Shigella sonnei, Salmonella typhimurium, Vibrio parahaemolyticus, Staphylococcus aureus, Bacillus cereus*, *Candida albicans*, *Candida tropicalis*, *Streptococcus agalactiae,* and *Bacillus thuringiensis*), and no template control (NTC). **(b)** Melting curve analysis of the O-CDA products by real-time PCR.

**Figure 7:**
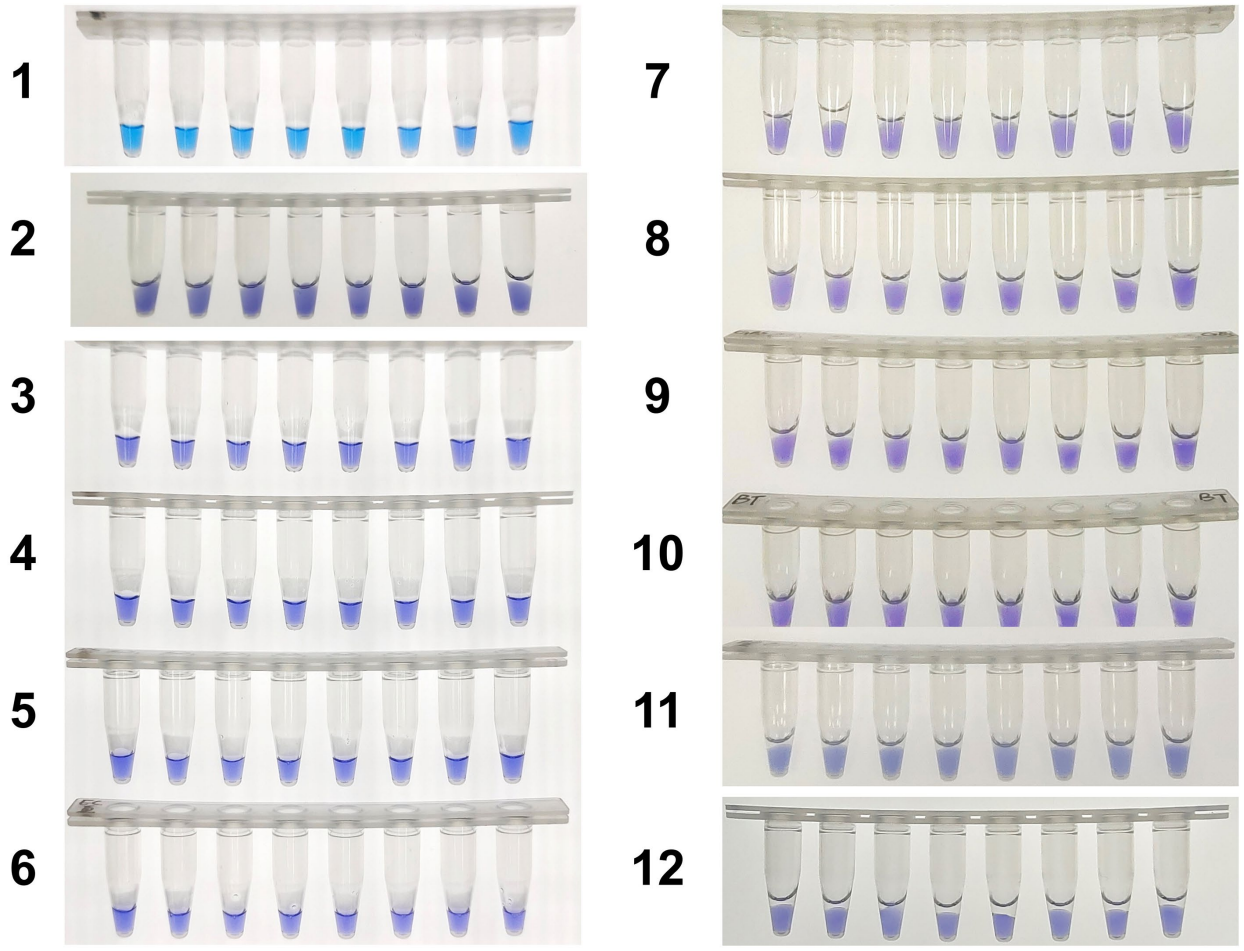
Colorimetric *hly*-O-CDA assay using HNB. Line 1, positive controls (10^5^ copies of templates, sky blue); Line 2, DNA samples extracted from *Listeria innocua* (violet); Line 3, DNA samples extracted from *Vibrio parahaemolyticus* (violet); Line 4, DNA samples extracted from *Shigella sonnei* (violet); Line 5, DNA samples extracted from *Salmonella typhimurium* (violet); Line 6, DNA samples extracted from *Escherichia coli* (violet); Line 7, DNA samples extracted from *Staphylococcus aureus* (violet); Line 8, DNA samples extracted from *Bacillus cereus* (violet). Line 9, DNA samples extracted from *Candida albicans* (violet). Line 10, DNA samples extracted from *Candida tropicalis* (violet). Line 11, DNA samples extracted from *Streptococcus agalactiae* (violet). Line 12, DNA samples extracted from *Bacillus thuringiensis* (violet).

Moreover, the specificity and sensitivity of this diagnostic system was determined after the evaluation of 560 batches of DNA samples, which were extracted from 11 different origins *L. monocytogenes* strains, artificially contaminated materials by standard *L. monocytogenes* strain (CVCC 1598), and other bacteria strains, as compared to standard method (Table 2). The results indicated that the primers of *hly* O-CDA method specifically identified and amplified the targeted DNA sequence of 208 batches of 11 standard *L. monocytogenes* strains samples and 40 batches of artificially contaminated samples and 16 batches of samples collected from refrigerators (Figure 6a). The melting curve analysis showed the Tm values of 78.20°C (Figure 6b); no difference was observed between positive controls and different DNA samples. Furthermore, to evaluate the feasibility of the *hly* O-CDA method, 40 batches of DNA extracted from contaminated samples containing different concentrations of *L. monocytogenes* were tested. The results confirmed that the newly developed O-CDA assay could effectively detect *L. monocytogenes* in samples of different origins. Furthermore, the *hly* O-CDA method monitored by real-time fluorescence curve and endpoint colorimetric observation was in coincidence (Figures 6, 7). Overall, *hly* O-CDA detection achieved a high sensitivity (100%), specificity (100%), and accuracy (Table 2).

**Table 2:** Determination of sensitivity and specificity of O-CDA assay for *Listeria monocytogenes*.

Species	Sample numbers		Sensitivity	Specificity	Accuracy
	O-CDA	Defined	95% CI *	95% CI *	95% CI *
*Listeria monocytogenes*	208	208	1.0 (98.2–100.0)	1.0 (99.0–100.0)	1.0 (99.3–100.0)
*Listeria innocua*	0	32			
*Escherichia coli*	0	32			
*Shigella sonnei*	0	32			
*Salmonella typhimurium*	0	32			
*Vibrio parahaemolyticus*	0	32			
*Staphylococcus aureus*	0	32			
*Bacillus cereus*	0	32			
*Candida albicans*	0	32			
*Candida tropicalis*	0	32			
*Streptococcus agalactiae*	0	32			
*Bacillus thuringiensis*	0	32			
Total	208	560			

* CI: confidence interval. Statistical analysis was carried out by an online program of “Diagnostic test evaluation calculator,” https://www.medcalc.org/calc/diagnostic_test.php.

## 4 Discussion

Targeted specific gene signal recognition and amplification by real-time qPCR have been taken as regular approaches to provide quantitative and semi-quantitative DNA and RNA biomarker analysis (Ding et al., 2023). PCR-based *in vitro* diagnosis kits permitted by the governments have played vital roles in the control of the SARS-CoV-2 pandemic (Lu et al., 2020; Song et al., 2021). Regretfully, current PCR procedures rely on standard laboratories, real-time PCR equipment, and well-trained qualified operators, which are relatively of high-cost and time-consuming (Tong et al., 2024). Furthermore, it is hard for standard PCR-based nucleic acid diagnosis to be adopted in undeveloped regions for sudden airborne and foodborne disease outbreaks (Lim et al., 2024).

Isothermal amplification assays, such as LAMP, have emerged as promising lab-independent and at-home point-of-care test alternatives to RT-qPCR (Wilner et al., 2023). In this study, we have developed assays for the detection of *L. monocytogenes* based on newly established CDA with simple primer design and low primer cost compared with LAMP (Figure 1b). The CDA method was successfully developed and optimized with a set of primers to amplify *L. monocytogenes* DNA. Both real-time fluorescence and visual-based *L. monocytogenes* CDA detection methods showed good repeatability and stability, indicating the assays would be effective and specifically identify *L. monocytogenes* strains.

Performance of the *L. monocytogenes* O-CDA assay, i.e., detection limit, sensitivity, and specificity, was determined. The detection limits of both real-time and endpoint visual O-CDA were similar to those of real-time PCR and were determined to be 1 copy/μl. The cycle threshold (Ct) value of 1 copy *L. monocytogenes* gene was 47 min, exhibiting high efficiency of the developed method. However, we failed to obtain a fine standard amplification curve of the developed CDA assay, suggesting its limited potential for quantitative analysis.

In the previous studies of LAMP and CAMP methods, the detection limits for *L. monocytogenes* were 2.82 × 10^2^ CFU/mL based on the phosphatidylcholine-phospholipase C gene (Wachiralurpan et al., 2018) and 1.0 × 10^2^ CFU/mL based on the *hly*A gene (Li et al., 2020), which were higher than that of the *hly* CDA method. Moreover, in RPA based methods, the detection limits for *L. monocytogenes* were 2 × 10^4^ cells/mL using an integrated microfluidic chip (Eid and Santiago, 2017) and 1 CFU/mL using a lateral flow strip (Wang et al., 2020). Another RPA-LFD method established by Mabrok achieved a detection limit of 200 fg total DNA and 0.4 CFU (Mabrok et al., 2021). As to the newly developed polymerase spiral reaction (PSR) based method, the sensitivity of *L. monocytogenes* was determined to 5.1 × 10^1^ CFU/g of artificially contaminated fruit (Zhang M. R. et al., 2024). The CDA method could achieve a similar detection limit with less cost compared with the expensive RPA-based approach, as fewer enzymes and shorter primers were needed. The application of CDA other than the isothermal amplification method would lead to a dramatic cost saving in primer expense (approximately 25%) compared with LAMP, CAMP, and PSR methods. The specificity tests have demonstrated that the developed *hly* O-CDA assays in this study can discriminate *L. monocytogenes-*related microorganisms such as *Vibrio parahaemolyticus, Shigella sonnei, Salmonella typhimurium, Escherichia coli, Staphylococcus aureus*, *Bacillus cereus*, *Candida albicans*, *Candida tropicalis*, *Streptococcus agalactiae,* and *Bacillus thuringiensis.* Furthermore, the detection procedures and time needed for the CDA assay were much shorter than the real-time qPCR.

In conclusion, the real-time fluorescence and visual-based *L. monocytogenes* O-CDA detection methods were established, both offered 100% sensitivity and specificity after optimization and verification. The real-time fluorescence plots of *L. monocytogenes* DNA sample amplification and following melting curve analysis-based *hly* O-CDA were suitable for laboratory-based accurate diagnosis. Considering the portable manipulation of the HNB-based colorimetric detection system, our results shed light on its potential application for on-site *L. monocytogenes* monitoring. The developed CDA-based methods are rapid, simple, reliable, and sensitive in several samples, showing potential to manage the task of *L. monocytogenes* infection warning more easily. It is expected that the developed *hly* O-CDA assay could evolve into a powerful point-of-care diagnostic platform when combined with microfluidics (Land et al., 2019).

## Data Availability

The datasets presented in this study can be found in online repositories. The names of the repository/repositories and accession number(s) can be found in the article/Supplementary material.

## Footnotes

^1^ https://www.thermofisher.cn/cn/zh/home/brands/thermo-scientific/molecular-biology/molecular-biology-learning-center/molecular-biology-resource-library/thermo-scientific-web-tools/dna-copy-number-calculator.html
