# Supplementary material for: Rapid and simple detection of Listeria monocytogenes using real closed dumbbell-mediated isothermal amplification
Source: Front Microbiol. 2025 Aug 19;16:1596797. doi: 10.3389/fmicb.2025.1596797 (PMC12401984; doi:10.3389/fmicb.2025.1596797)
Supplement: Supplementary file 1 [file Table_1.DOCX]

Supplementary Material

**Rapid and simple detection of *Listeria monocytogenes* using closed dumbbell mediated isothermal amplification (CDA)**

**Yanli Zhang ^1, +^, Xinyao Wu ^2, +^, Yeling Zhong ^3^, Xuhan Chen ^2^, Fei Guo ^4^, Guifang Ouyang ^1, *^, and Rui Mao ^2, *^**

^1^ Department of Hematology, The First Affiliated Hospital of Ningbo University, Ningbo 315010, Zhejiang, China

^2^ Ningbo Institute of Life and Health Industry, University of Chinese Academy of Sciences, Ningbo 315010, China.

^3^ Department of General Surgery (Hepatic, Anal-canal, Gastrointestinal), Ningbo Zhenhai People’s Hospital, Ningbo 315020, Zhejiang, China.

^4^ Department of Laboratory Medicine, The First Affiliated Hospital of Ningbo University, Ningbo 315010, Zhejiang, China.

*** Correspondence:**

Guifang Ouyang

fyyouyangguifang@nbu.edu.cn

Rui Mao

[mr3749@163.com](mailto:mr3749@163.com)

^+^ These authors contributed equally to this work

# Supplementary Tables

###### Supplementary Table 1 Four pairs of LM-CDA primers were designed by DNAMAN

| Pirmer | Sequence |
| --- | --- |
| LM-MF-1 | TACCGTCGATGACGTAAGTGGGAAATCTGTCT |
| LM-MR-1 | ACCTCGGAGACTAATGGGAACTCCTGGTGT |
| LM-MF-2 | TTCTGCATTCACAACCTACAAGACCTTCC |
| LM-MR-2 | AATCCTCCTGCAACCTGAGACAGATTTCC |
| LM-MF-3 | ACGGCTTTGAAGGATCTGTCTCAGGTGATG |
| LM-MR-3 | AATTTACGGTGGTATCTCGTAAGTCTCCG |
| LM-MF-4 | TTACCGTTCTCCATATTTGCCAGGTAACGC |
| LM-MR-4 | ATTGATGACCGGATCGGATAAAGTGTAGTGCC |

###### Supplementary Table 2 Bacterial strains used in this study

| Species | Strain | Source | Year |
| --- | --- | --- | --- |
| *Listeria monocytogenes* | CVCC 1597 | CVCC | 2021 |
| *Listeria monocytogenes* | CVCC 1598 | CVCC | 2021 |
| *Listeria monocytogenes* | CVCC 1599 | CVCC | 2021 |
| *Listeria monocytogenes* | CGMCC 1.10753 | CGMCC | 2021 |
| *Listeria monocytogenes* | CGMCC 1.9144 | CGMCC | 2021 |
| *Listeria monocytogenes* | CGMCC 1.9136 | CGMCC | 2021 |
| *Listeria monocytogenes* | BNCC 336877 | BNCC | 2021 |
| *Listeria monocytogenes* | BNCC 314197 | BNCC | 2021 |
| *Listeria monocytogenes* | ATCC 19115 | ATCC | 2021 |
| *Listeria monocytogenes* | SAU-LM001 | FSC_SAU | 2021 |
| *Listeria monocytogenes* | SAU-LM001 | FSC_SAU | 2021 |
| *Listeria innocua* | ATCC 33090 | ATCC | 2025 |
| *Vibrio parahaemolyticus* | CGMCC 1.1997 | CGMCC | 2021 |
| *Shigella sonnei* | CVCC 3926 | CVCC | 2020 |
| *Salmonella typhimurium* | ATCC 14028 | ATCC | 2020 |
| *Escherichia coli* | CGMCC 1.12883 | CGMCC | 2021 |
| *Staphylococcus aureus* | CGMCC 1.6750 | CGMCC | 2021 |
| *Bacillus cereus* | CICC 21261 | CICC | 2020 |
| *Candida albicans* | CICC 1965 | CICC | 2021 |
| *Candida tropicalis* | BNCC 186815 | BNCC | 2021 |
| *Streptococcus pyogenes* | BNCC 185941 | BNCC | 2022 |
| *Bacillus thuringiensis* | BNCC 133175 | BNCC | 2022 |
